# Effectiveness of ultrasound‐guided removal of intrauterine devices

**DOI:** 10.1111/ajo.13584

**Published:** 2022-07-11

**Authors:** Lynn Townsend, Elizabeth Luxford, Karen Mizia

**Affiliations:** ^1^ SAN Ultrasound for Women, Sydney Adventist Hospital Sydney New South Wales Australia; ^2^ School of Women and Children’s Health University of New South Wales Sydney New South Wales Australia; ^3^ ANU Medical School Australian National University College of Health and Medicine Sydney New South Wales Australia; ^4^ Maternal Fetal Medicine Unit Royal North Shore Hospital Sydney New South Wales Australia

**Keywords:** intrauterine device, removal, ultrasound guidance

## Abstract

A retrospective review over 12 months was conducted to assess the success rates for ultrasound‐guided removal of an intrauterine device (IUD). Cases were broken up into two groups. There were 241 cases in which removal had been unsuccessful in the office setting. Where the strings were not visible the device was successfully removed in 97.7% of attempts. The second group had visible strings and 100% were successfully removed. The success rate for ultrasound‐guided removal of IUDs when the strings are not visible supports the pilot study published in 2012. This approach can successfully avoid the need for sedation and or hospital admission in over 97% of cases.

In 2013, Mizia and Ramsay reviewed the successfulness of ultrasound‐guided procedures for removal of an intrauterine device (IUD). In this initial pilot study they reviewed 38 cases which were referred where the strings were not visible and office procedures had failed to remove the device.^1^ Of these, 33 were successful, giving a success rate of 86.8%.

Since 2013, there have been few articles published.^2^, ^3^, ^4^, ^5^, ^6^ One of the largest cohorts was published by Verma^4^ who reviewed the safety and cost‐effectiveness of ultrasound‐guided removal of retained IUDs in a small number of women. In a small series of 23 cases 83% were successfully removed.

The objective of this paper is to review the rate of success of cases referred for ultrasound‐guided removal of an IUD and review the success rate of ultrasound‐guided attempts for removal when the strings are not visible and office procedures have failed over a 12 month period.

## Materials and Methods

This is a retrospective cohort study of consecutive women referred for ultrasound‐guided removal of an IUD. We then analysed the success for removal. Cases were performed across eight Sydney practices between the 1 January 2020 and 31 December 2020. We then divided these cases into two groups. The first group consisted of cases where the strings were not visible, and attempts at removal in the office setting had failed. The second group consisted of cases who were referred for removal where the strings were visible. Data regarding the indication for referral were collected.

Equipment utilised for IUD removal included fine‐toothed forceps, 3 mm balloon‐tip catheters, plastic retrieval devices, intracavity lignocaine and intracavity normal saline. Device retrieval was performed under aseptic technique, with vaginal speculum, adequate lighting and with transabdominal scanning by a separate clinician. Patients were requested to present with a comfortably full bladder. This optimised imaging guidance and potentially reduced the angle of the cervical‐corpus junction All procedures were performed by a specialist obstetrician gynaecologist.

Approval for collection of data and publication was granted by the Sydney Adventist Hospital Institutional Ethics Committee.

## Results

Two hundred and forty‐one women presented for removal of an IUD between January and December 2020. One hundred and seventy‐three patients were referred where strings were not visible following unsuccessful attempts of removal by their practitioner. The methods used in the original attempted removal were not disclosed. Ten of these cases involved a copper IUD, one involved a Jaydess device and one was of uncertain origin. The remainder were Mirena IUDs. The average age of the patient was 41.6 years with a range of 22–59 years. The device was successfully retrieved in 169 cases, giving a 97.7% success rate. Of these, 20 women were primigravid, 49 had at least one child and in 100 cases, parity was not recorded.

Of the four cases where ultrasound‐guided attempts failed, one case was abandoned due to patient discomfort; the remaining three cases were unsuccessful despite the use of multiple techniques. All four devices were successfully removed under general anaesthetic.

In comparison, the average age of the 68 patients referred for ultrasound‐guided removal in which the strings were visible was 40.5 years with a range of 24–57 years. Nine of these cases involved a copper IUD, two were Kyleena devices with the remainder involving Mirena IUDs. Of these, 20 women were primigravid, 29 women had at least one child and parity was not recorded in the remainder. There were no cases of unsuccessful retrieval in the group where the strings were visible. Table 1 demonstrates the aforementioned results. Of cases in which strings were visible at the time of removal, the indication for referral to our clinics included replacement, patient or clinician preference, malposition, simultaneous pre‐conception review, patient anxiety, outside referrer scope of practice and inability to perform in the referrer rooms. Table 2 demonstrates this data breakdown.

**Table 1:** Success rates for removal of an intrauterine device with strings not visible compared with strings visible

	Strings not visible	Strings visible
Total number	173	68
Age of patient, mean years (range)	41.6 (29–46)	40.5 (23–56)
Successful removal (%)	169 (97.7)	68 (100)

**Table 2:** Referral indications and specialist breakdown

Case group	Indication for referral	No. of patients (% of total group)	Referral source	No. of patients (% of total group)
Strings not visible	Unable to remove	173 (72)	General practitioner	148 (86)
			Obstetrician gynaecologist	25 (14)
Strings visible		68 (28)	General practitioner	48 (71)
			Obstetrician gynaecologist	19 (28)
			Other specialist	1 (1)
		No. of patients (% of strings visible group)		
	Anxiety	3 (5)		
	Outside scope of practice	1 (1)		
	Pre‐conception	4 (6)		
	Preference	12 (17)		
	Malposition	4 (6)		
	Replacement	42 (62)		
	Unable in rooms	2 (3)		

Patients were referred by obstetrician gynaecologists, general practitioners and other specialists. Indication for referral is broken down by specialist group in Table 2.

There were no cases with significant vasovagal symptoms and no woman reported concerning bleeding, pain or infective symptoms in either group.

## Discussion

Our data show that ultrasound‐guided removal of an IUD when the strings are not visible and office attempts had been unsuccessful is highly effective. In 2013, ultrasound guidance was success in retrieving 83% of devices,^1^ whereas in 2020 the success rate is 97%. While the studies cannot demonstrate comparative matched patient populations, they reflect a high degree of success in achieving the desired clinical outcome. This may reflect improvements in clinician skill and/or ultrasound technology. Further studies utilising matched population data would be of value in corroborating these findings. Having demonstrated the success of IUD removal under ultrasound guidance, the study demonstrates how the majority of patients have potentially avoided the need for anaesthesia, theatre time, and time off from usual activities.

Although this is a retrospective review, this is the largest cohort reported in the literature. There are several small cases series,^2^, ^3^, ^4^, ^6^ the largest of which was published by Verma^4^ involving 23 women, and a poster^7^ presenting similar success rates to this study.

## Conclusion

Overall, this study confirms the findings of the 2013 pilot study and has shown that ultrasound‐guided removal of an IUD when the strings are not visible and office settings have failed is successful in the majority of cases. It would be reasonable to offer ultrasound guidance as a first line procedure when the strings are not visible, avoiding the need for anaesthetics in most cases.
